# Preparation of Quick-Dissolving Nanofiber Face Masks Based on Needleless Electrostatic Spinning

**DOI:** 10.3390/polym16111602

**Published:** 2024-06-05

**Authors:** Jingyi Hu, Xiaojie Chen, Jianmin Jiang, Renbiao Mai, Han Wang, Qiming Xu, Ting Zhang

**Affiliations:** 1 State Key Laboratory of Precision Electronic Manufacturing Technology and Equipment, Guangdong University of Technology, Guangzhou 510006, China; 15107687483@163.com (X.C.); 17301991036@163.com (J.J.); 18665139415@163.com (R.M.); wanghangood@gdut.edu.cn (H.W.); 18551788821@163.com (Q.X.); zhang_ting@gdut.edu.cn (T.Z.); 2 School of Electromechanical Engineering, Guangdong University of Technology, Guangzhou 510006, China

**Keywords:** needleless, facial mask, electrospinning, nanofiber

## Abstract

As the global facial mask market continues to grow, consumers have put forward higher requirements for the functionality and ingredients of mask products. Ordinary facial masks mostly use ordinary non-woven fabrics as the mask base fabric and are used with essence. Preservatives are generally added. At the same time, they are susceptible to the influence of the external environment and are easily oxidized, causing the mask to deteriorate and cause skin allergic reactions. In addition, traditional facial masks have problems such as poor fit with the skin, poor breathability, insufficient absorption of nutrient solutions, and easy dripping. The high specific surface area and high porosity of a nanofiber mask prepared by electrospinning technology are beneficial to the skin’s absorption of nutrients, and it has good fit with the skin and strong breathability. A unique advantage of this nanofiber mask is that it uses spray. After the mask is sprayed with water or essence, the water-soluble polymer within it can be quickly dissolved, saving a lot of time. Nanofiber facial mask products can effectively solve consumer pain points and are conducive to the high-end development of facial masks. Therefore, this article combines needleless electrospinning technology to develop a new solid-state, preservative-free, quick-dissolving nanofiber facial mask that can be prepared on a large scale. Based on needleless electrospinning technology, this article deeply explores the process parameters and their influencing mechanisms for preparing nanofiber, quick-dissolving facial masks to achieve the stable preparation of nanofiber facial masks with the best morphology; a comprehensive analysis of the structure and influence of nanofiber facial masks from micro and macro perspectives demonstrates their performance and allows evaluation of them. The experimental results show that the mask morphology is optimal under the process conditions of using a spinning liquid of 20% collagen peptide solution, a spinning voltage of 30 kV, a collection distance of 19 cm, and a liquid supply speed of 130 mL/h.

## 1. Introduction

At present, most of the conventional masks on the market use ordinary non-woven fabrics as base fabrics with essence. However, some unstable active ingredients in the liquid components of facial masks are easily oxidized and deteriorated by the external environment, causing skin allergies and other problems [1]. Electrospinning, an emerging facial mask fabrication technology, can generate nanofiber masks that effectively tackle the previously mentioned issues [2]. This technique allows for the production of continuous nanoscale fibers derived from both synthetic and natural polymers [3,4,5]. The fibers generated by electrostatic spinning have the characteristics of high porosity, large specific surface area, high air permeability, and small pore size within the fiber [6,7]. Therefore, electrospinning has received widespread attention and has been used in tissue engineering [8], filtration [9], energy [10], sensors [11], food engineering [12], and cosmetics [13]. As a novel type of facial mask, there are relatively few nanofiber mask products available on the market [14]. With the increasing market demand for high-end facial mask products, the development of a nanofiber facial mask amenable to large-scale production via electrospinning technology holds great significance. Nanofiber masks based on electrospinning technology can efficiently capture active ingredients within fibers or meshes. Moreover, the nanofiber mask is stored in a solid-state and will absorb water and become moist when applied to the skin, which greatly enhances product stability. When the facial mask becomes wet, the components within it rapidly dissolve and release active ingredients, ensuring maximum penetration into the skin and restoring its youthful state. In addition, the nanostructure of the nanofiber mask can ensure maximum fit with the skin surface. The nanofiber mask is not only softer than traditional masks, but its nutrients are also more readily released onto the skin’s surface, enhancing skin permeability and restoring its youthful state. According to different consumer needs, different raw materials can be used to produce nanofiber masks with different functions through electrospinning technology to achieve personalized care and precise skin care. Currently, many different types of nanofiber membranes have been produced based on electrospinning technology. ActiVLayr nanofiber technology has prepared a quick-dissolving nanofiber mask. This mask is made from deep-sea puffer fish in New Zealand. It contains a variety of natural collagen extracts and active ingredients. It will quickly dissolve in just 3 to 5 s and is ready for use. It offers an extremely fast skin care experience [15,16]. In addition, nanofibers can load a variety of cosmetic ingredients, such as glycerin, vitamin C, okra polysaccharides, active polypeptides, tea polyphenols, and small-molecule sodium hyaluronate. When the quick-dissolving nanofiber mask comes into contact with water, these functional ingredients will quickly be absorbed by the skin. Yang et al. [17] prepared a multifunctional cellulose mask containing hindered phenol groups through electrospinning technology, which has excellent free radical scavenging properties and can effectively remove excessive free radicals while shielding ultraviolet damage and delaying the aging of skin tissue. Tang et al. [18] provide a facile approach to develop biocompatible, polymer-based electrospinning fiber masks to effectively deliver herbal extracts for topical skin treatment, and it has shown significant clinical efficacy in the treatment of mild to moderate acne, opening up new prospects for a new generation of beauty textiles.

Therefore, in order to solve the problems of traditional facial masks such as poor comfort, poor breathability, insufficient absorption of nutrient solution, and solution dripping, this paper develops a high-performance nanofiber facial mask through needleless electrospinning technology. We chose a mixed solution of polyethylene oxide and fish collagen peptide as the spinning solution. Fish collagen peptide is a type of collagen extracted from fish skin or fish scales. It has a variety of effects and functions, especially in beauty. Fish collagen peptide can maintain skin moisture because it contains hydrophilic natural moisturizing factors, which can effectively lock in moisture, nourish the skin, prevent skin wrinkles, enhance the activity of skin collagen, enhance blood circulation, shrink pores, and cause fine lines to disappear [19,20,21]. Polyethylene oxide (PEO) can be mixed with facial mask essence to form a spinning solution, which has excellent spinning assistance ability. PEO is a crystalline and thermoplastic water-soluble material that is completely soluble in water and has high solution viscosity. Different from other polymers, PEO has excellent properties such as low cost, non-toxicity, good solution rheology, and complete water solubility. The solution has good spinnability [22], and the finished membrane nanofiber diameter after spinning is uniform, evenly distributed, with a large specific surface area and good pore structure, which can greatly improve the carrying capacity of the active ingredients in the mask, and has strong water retention and good biocompatibility [23,24]. This article also explores the optimal preparation process of nanofiber facial masks, comprehensively analyzes the performance of nanofiber facial masks, and highlights their advantages as a new type of nanofiber facial mask.

## 2. Materials and Methods

Collagen peptide powder for the experiment was sourced from Shandong Xinsheng Biotechnology Co., Ltd. in Dongying City, China, while polyethylene oxide (PEO) with a molecular weight of 500,000 was obtained from Shanghai Aladdin Biochemical Technology Co., Ltd. in Shanghai, China. Non-woven fabrics were purchased from Shijiazhuang Tianjinsheng Nonwoven Technology Co, Ltd. in Shijiazhuang City, China. Deionized water was purchased from Aite (Shandong) New Materials Co, Ltd. in Jinan City, China. At room temperature, PEO was combined with deionized water to create a transparent solution with a mass fraction of 9% *w*/*v*. An electric stirrer was used to stir for 6 h until the solution was uniform. Then, the collagen peptide powder and deionized water solvent were mixed to form a facial mask essence solution with a mass fraction of 10% *w*/*v* and stirred at room temperature for 4 h until the solution was uniform. Finally, the prepared PEO solution and collagen peptide essence were mixed to make 5 groups of solutions. The proportions of collagen peptide solution in PEO solution were 0%, 10%, 15%, 20%, and 25%, respectively. Each set of solutions was thoroughly stirred for 4 h until uniform and transparent, which was used as the spinning solution.

## 3. Preparation of Nanofiber Facial Mask

**Figure 1:**
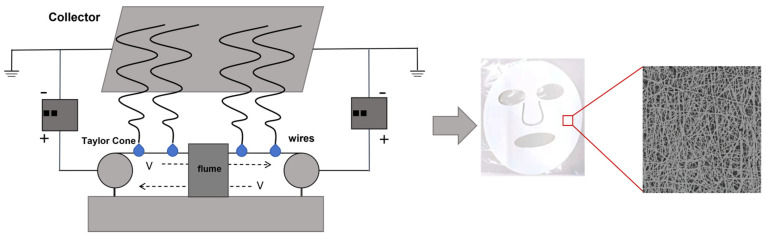
Preparation process of nanofiber mask.

The needleless electrospinning device mainly consists of a liquid supply system, a high-voltage power supply, a metal wire, and a collection platform. Its working principle is shown in Figure 1. Before preparing the nanofiber mask, wind the steel wire tightly onto the spinning electrode. Turn on the environmental control module of the device. Wait for two hours. Adjust the humidity to (40 ± 2)%, and the temperature to (26 ± 2) °C. When the temperature and humidity inside the spinning equipment are stable, fit the base material to the collection platform and adjust the appropriate liquid supply speed. The receiving speed is 0.5 m/min. Pour the spinning liquid of PEO/collagen peptide essence into the liquid tank, and evenly apply the spinning liquid to the steel wire through the reciprocating motion of the liquid tank. After the steel wire is wetted, a uniform solution film is formed on the surface. Apply a high-voltage electrostatic field between the steel wire electrode and the receiving device. When the charge density on the surface of the solution thin layer reaches a critical value, the electrostatic force overcomes the surface tension of the solution and the solution is stretched. A jet is formed and ejected. As the jet flies toward the receiving device, it undergoes solvent evaporation and stretching, and finally deposits on the receiving device to form nanofibers. After spinning for a period of time, collect the nanofiber masks of a certain thickness formed by different process parameters (voltage, liquid supply speed, collection distance) by adjusting the process parameters. The preparation process is shown in Figure 1. Finally, cut and place the prepared nanofiber mask in a drying box for 6 h, dry it at room temperature, and then seal and store it.

## 4. Characterization Method

First, the nanofiber facial mask prepared by the needleless electrospinning device was cut and sampled in a targeted manner, and then a plasma thin-film sputtering instrument (GSL1100X-SPC-12, Hefei Kejing Material Technology Co., Ltd., in Hefei, China.) was used for gold spraying treatment, and then a Hitachi electron microscope equipment (TM4000, Shanghai Yiheng Scientific Instrument Co., Ltd., in Shanghai, China.) was used for detection. In order to prevent the fiber membrane from falling off during the vacuum extraction process, a mask sample of an appropriate size needs to be fixed on a metal specimen stage, and the fiber morphology magnified 1500 times was observed through an accelerating voltage of 5 kV. After taking the picture, ImageJ software (ImageJ 1.54g) was used to randomly select 50 fibers for measurement, and Origin software (Origin 2021) was used to count the fiber diameter distribution.

## 5. Results and Discussion

### 5.1. Effect of Different Mixing Ratios of PEO/Collagen Peptide Solutions on Fiber Morphology

In order to explore the effect of different ratios of PEO/collagen peptide solutions on fiber morphology, in this set of experiments, the liquid supply speed was 130 mL/h. The voltage was 30 kV. The collection distance was 19 cm, and the receiving speed was 0.5 m/min. Then, a PEO solution with a mass fraction of 9% *w/v* was prepared and mixed with collagen peptide essence solutions in different proportions. Set the collagen peptide solution proportions to 0%, 10%, 15%, 20%, and 25%, respectively. After spinning, 5 kinds of nanofiber facial masks with PEO/collagen peptide combinations were obtained.

**Figure 2:**
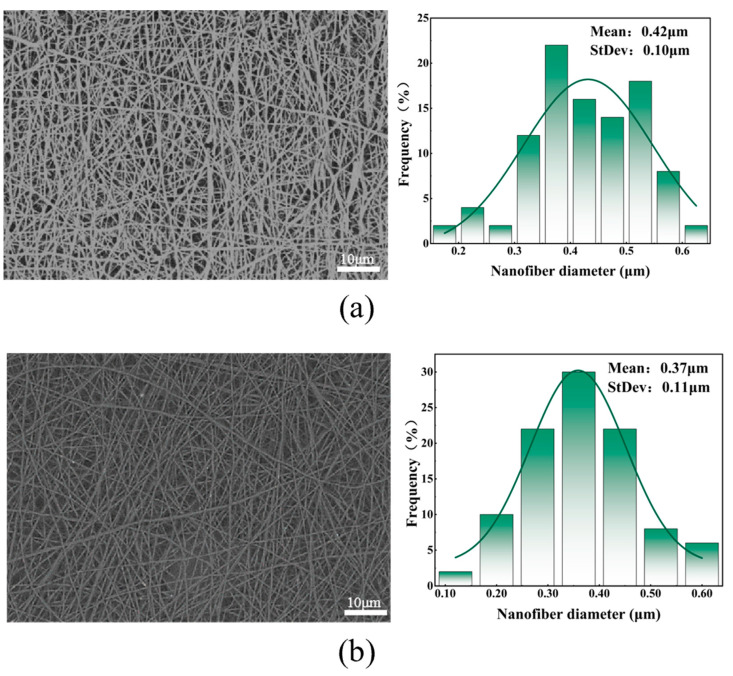

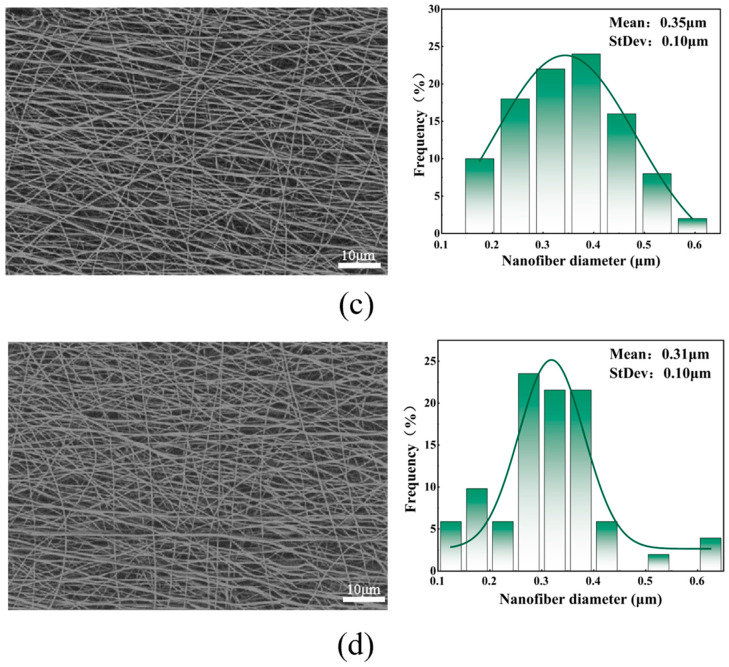

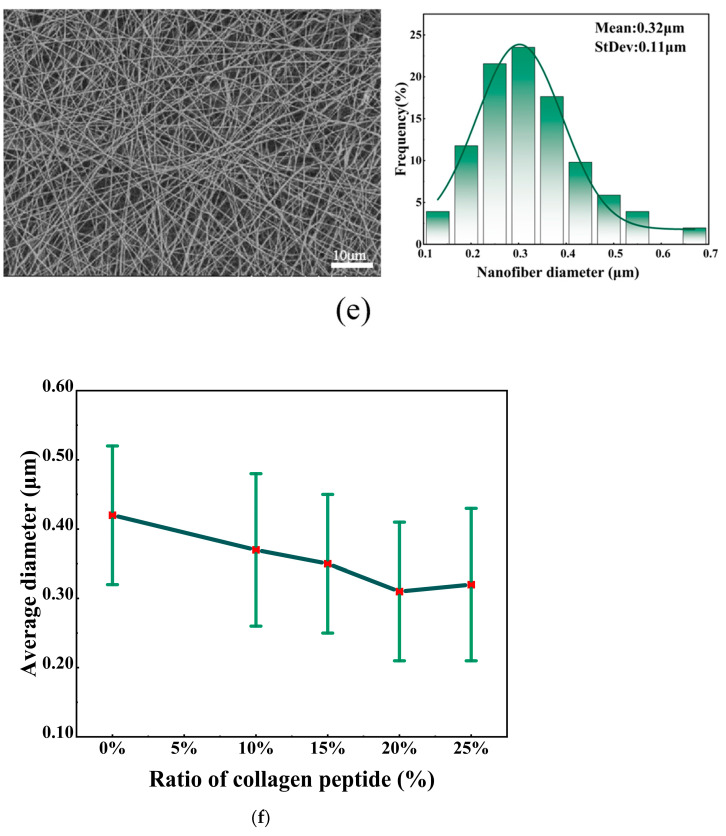
(**a**) SEM and diameter distribution diagram of facial mask fibers prepared with 0% collagen peptide solution; (**b**) SEM and diameter distribution diagram of facial mask fibers prepared with 10% collagen peptide solution; (**c**) SEM and diameter distribution diagram of facial mask fibers prepared with 15% collagen peptide solution; (**d**) SEM and diameter distribution diagram of facial mask fibers prepared with 20% collagen peptide solution; (**e**) SEM and diameter distribution diagram of facial mask fibers prepared with 25% collagen peptide solution; and (**f**) average fiber diameter of facial masks prepared with different collagen peptide solution ratios.

Figure 2 shows the SEM images, fiber diameter distribution statistics, and average fiber diameter of facial masks prepared from solutions with different collagen peptide proportions. Among the five collagen solutions with different ratios, when the proportion of collagen peptide solution was 0%, the average diameter of the fibers prepared was the largest. The fibers prepared with 20% collagen solution had the smallest average diameter, smaller standard deviation, and more concentrated nanofiber diameter distribution. This difference is mainly due to the viscosity characteristics of the spinning solution. As the proportion of collagen peptide solution increases, the viscosity of the spinning solution decreases, and the fibers are more obviously stretched during the forming process, causing the fibers to gradually become thinner. When the proportion of collagen peptide solution was increased to 25%, due to the low viscosity and insufficient intermolecular interaction, continuous fibers could not be formed and a fiber membrane with good morphology could not be obtained.

### 5.2. Effect of Different Spinning Voltages on Fiber Morphology

The formation of fibers requires exceeding a specific spinning voltage threshold. Only when the voltage threshold is exceeded can jets be generated and eventually refined into fibers. Therefore, voltage is identified as one of the key factors affecting fiber morphology. In order to explore the effect of voltage on fiber morphology, 20% collagen peptide solution was selected as the spinning solution. The liquid supply speed was set to 130 mL/h. The receiving speed was 0.5 m/min, and the voltage gradient was set to 20 kV, 25 kV, 30 kV, and 35 kV to further find the optimal voltage parameters. Presented below are nanofiber masks prepared under different voltages.

**Figure 3:**
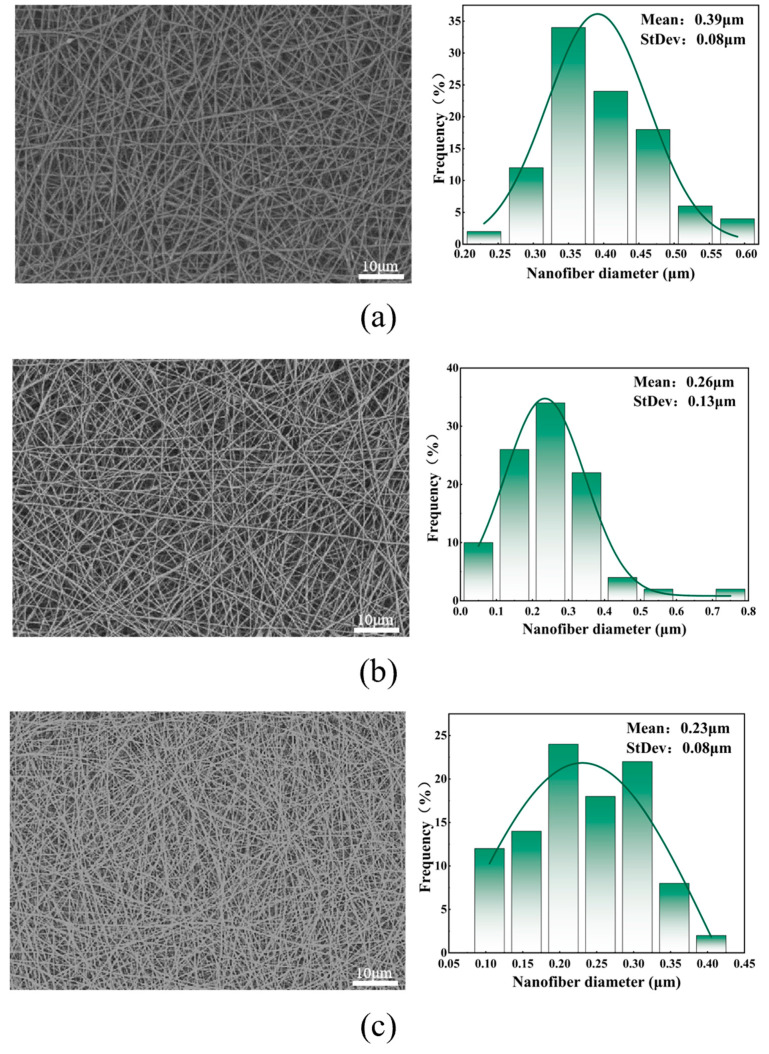

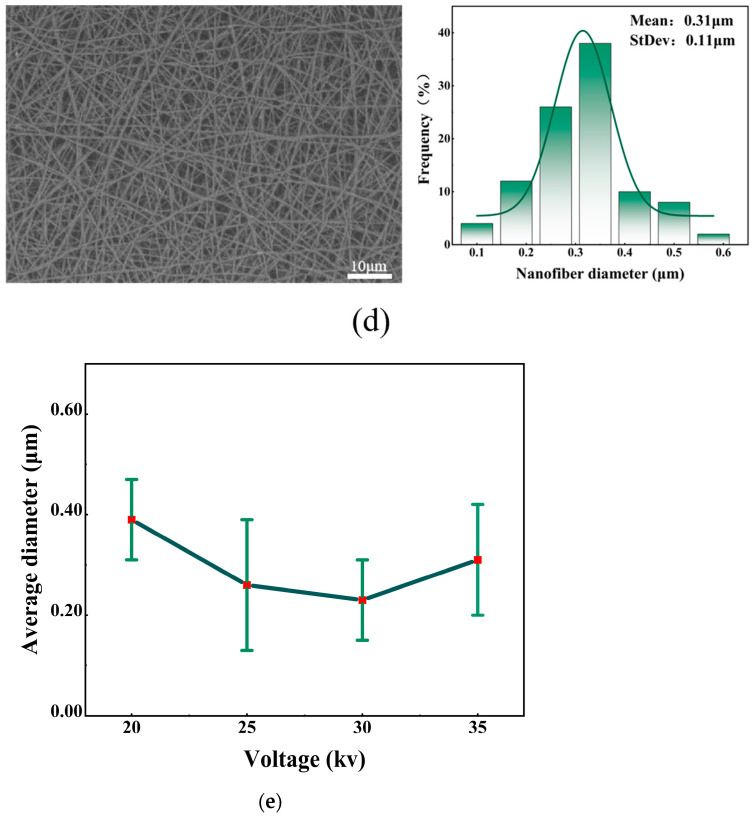
(**a**) SEM and diameter distribution diagram of facial mask fiber prepared at 20 kv voltage; (**b**) SEM and diameter distribution diagram of facial mask fiber prepared at 25 kv voltage; (**c**) SEM and diameter distribution diagram of facial mask fiber prepared at 30 kv voltage; (**d**) SEM and diameter distribution diagram of facial mask fibers prepared at 35 kv voltage; and (**e**) average fiber diameter diagram of facial masks prepared at different voltages.

Figure 3 shows the SEM images, fiber diameter distribution statistics, and average fiber diameter of the facial masks prepared under different voltage values measured. From the Figure 3, it can be seen that the electric field strength of electrospinning increases with the increase in applied voltage, causing the traction force of the multi-jet to increase, which in turn causes the fibers to become smaller and denser. However, when the voltage rises to a certain value, the traction force on the jet is further enhanced, increasing the instability of the jet, causing the average diameter of the fiber to begin to increase, and the standard deviation to become larger, resulting in uneven fiber distribution. Therefore, within the range of 20 kV to 35 kV, the voltage selection should avoid being too high or too low, so as not to cause the average diameter of the mask fibers to increase and the distribution to be uneven. The optimal voltage value is 30 kV. At this time, the average diameter and standard deviation of the fiber are smallest and the distribution is most uniform.

### 5.3. Effect of Different Receiving Distances on Fiber Morphology

Different receiving distances will affect solvent volatilization and multi-jet electric field intensity, resulting in changes in fiber morphology. In order to study the effect of receiving distance on fiber morphology, 20% collagen solution was used as a spinning solution. The liquid supply speed was set to 130 mL/h. The voltage was 30 kV. The receiving speed was 0.5 m/min, and the receiving distance gradient was set to 13 cm, 16 cm, 19 cm, and 22 cm to further determine the optimal collection distance. Below are the nanofiber masks at different collection distances.

**Figure 4:**
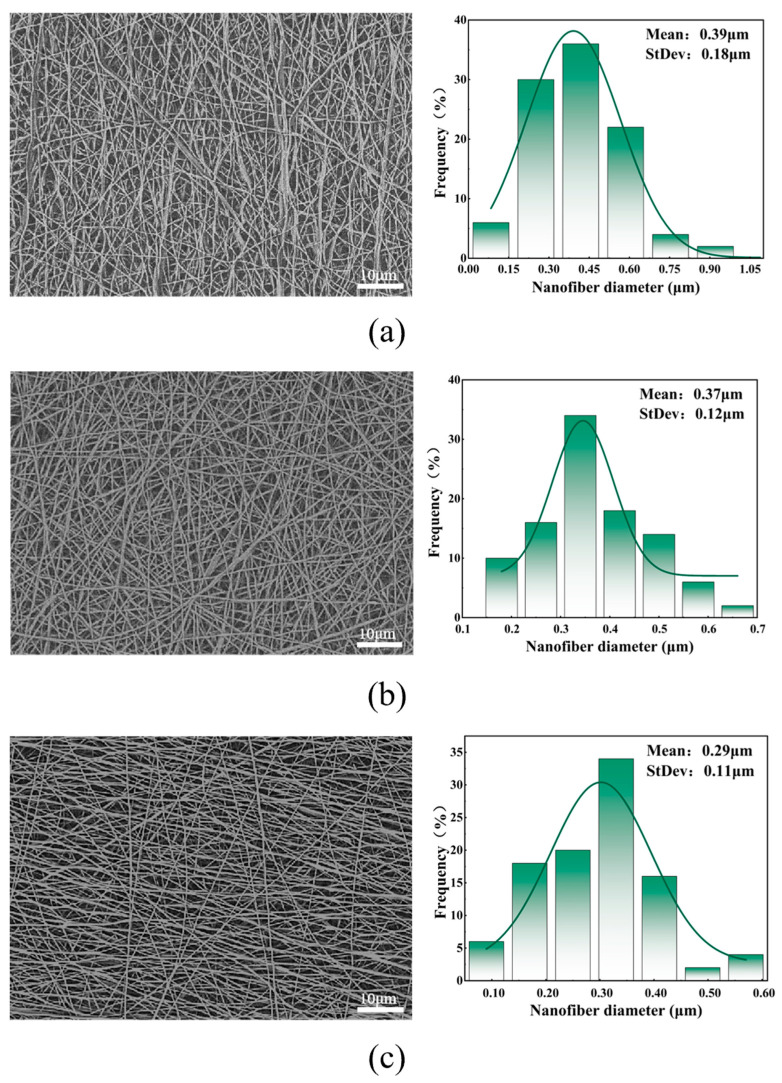

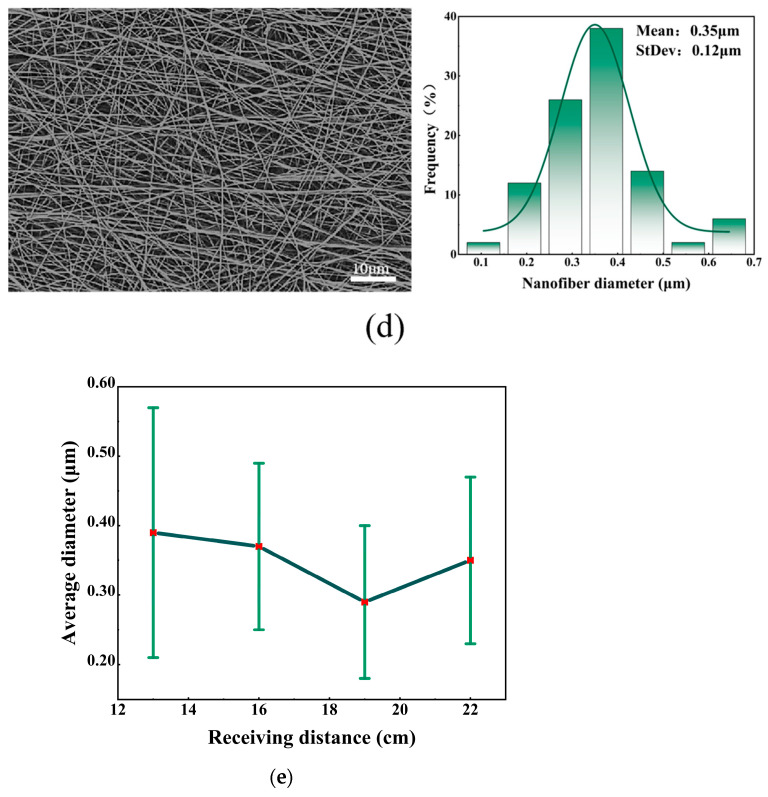
(**a**) SEM and diameter distribution diagram of facial mask fiber prepared with a receiving distance of 13 cm; (**b**) SEM and diameter distribution diagram of facial mask fiber prepared with a receiving distance of 16 cm; (**c**) facial mask fiber prepared with a receiving distance of 19 cm SEM and diameter distribution diagram; (**d**) SEM and diameter distribution diagram of facial mask fibers prepared with a receiving distance of 22 cm; and (**e**) average fiber diameter diagram of facial masks prepared with different receiving distances.

Figure 4 shows the SEM images, fiber diameter distribution statistics, and average fiber diameter of the facial masks prepared at different receiving distances. Combining the Figure 4, it can be seen that the morphology of the facial mask fiber is significantly affected by the receiving distance. This is because changes in the receiving distance cause changes in two key factors in the electrospinning process: the multi-jet electric field intensity and the degree of solvent volatilization. In this experiment, when the receiving distance was short, there were two factors acting simultaneously. When the electric field intensity was high, the traction force on the jet was sufficient, which resulted in a reduction in the flight time of the jet and insufficient solvent volatilization, thus making the diameter of the fiber larger. As the distance increases, the flight time of the jet gradually increases, the degree of solvent volatilization gradually increases, and the diameter of the fiber becomes thinner. However, when the receiving distance was far, the volatilization degree of the solvent did not change much. At this time, the electric field intensity becomes the dominant factor. When the electric field intensity of the multi-jet decreases, the traction force of the jet is insufficient, which in turn makes the diameter of the fiber larger.

Therefore, when conducting nanofiber mask experiments, the receiving distance needs to be adjusted according to the intensity of the jet electric field and the volatility intensity of the solvent to ensure that the best morphological fibers are obtained. The solvent used in this study is deionized water, which has high volatility, so a large receiving distance is required to fully evaporate the solvent. As can be seen from the above figure, when the receiving distance is 19 cm, the average diameter and standard deviation of the fibers are small and the distribution is relatively uniform, so 19 cm is the best receiving distance.

### 5.4. Effect of Different Liquid Supply Speeds on Fiber Morphology

The liquid supply speed will have a significant impact on the degree of solvent volatilization and preparation efficiency. Therefore, the liquid supply speed also plays a key role in needleless electrospinning. In order to explore the effect of liquid supply speed on fiber morphology, 20% collagen solution was selected as the spinning liquid, and the collection distance was set to 19 cm. The voltage was 30 kV, and the collection speed was 0.5 m/min. By setting the liquid supply speed gradient to 110 mL/h, 120 mL/h, 130 mL/h, and 140 mL/h, the optimal liquid supply speed was further found. In this study, a series of nanofiber facial masks at different liquid supply speeds were prepared to explore the effect of liquid supply speed on their performance.

**Figure 5:**
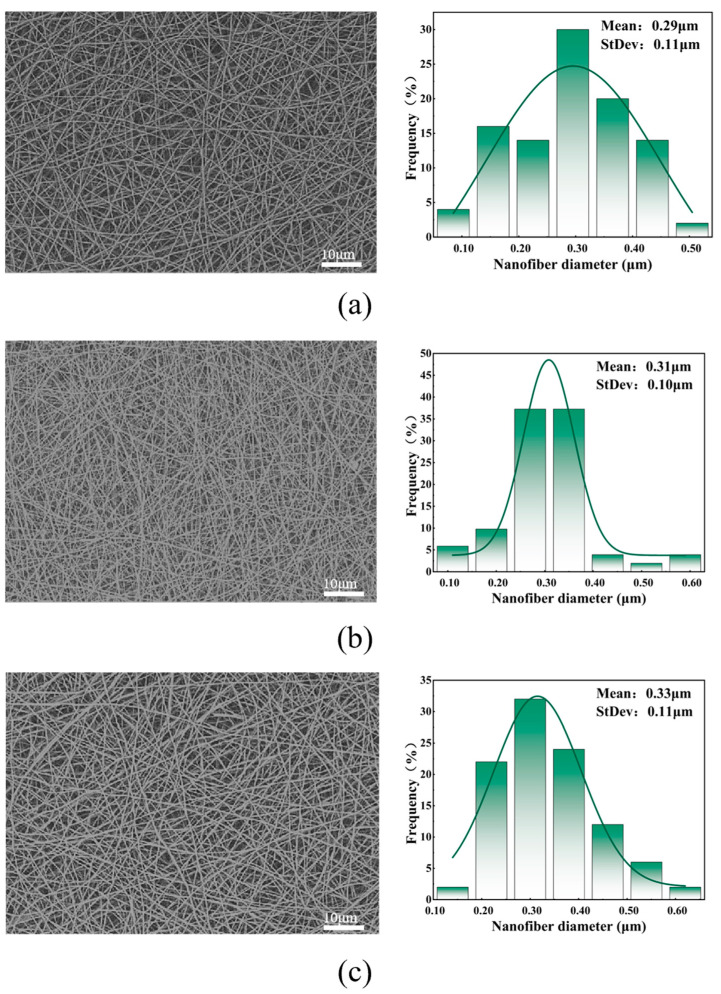

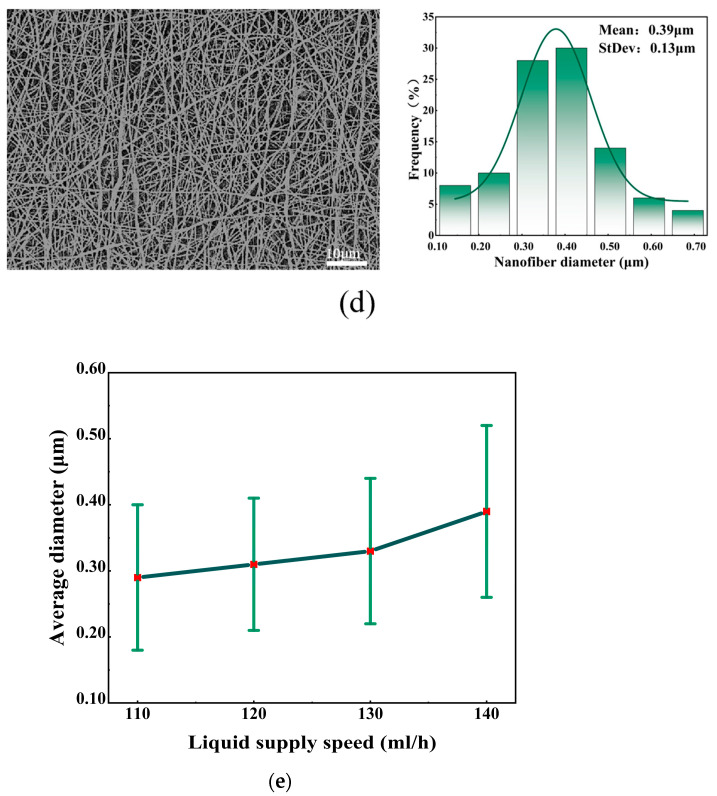
(**a**) SEM and diameter distribution diagram of facial mask fibers prepared with a liquid supply speed of 110 mL/h; (**b**) SEM and diameter distribution diagram of facial mask fibers prepared with a liquid supply speed of 120 mL/h; (**c**) liquid supply speed SEM and diameter distribution diagram of facial mask fiber prepared at 130 mL/h; (**d**) SEM and diameter distribution diagram of facial mask fiber prepared at 140 mL/h liquid supply speed; and (**e**) fibers of facial mask prepared at different liquid supply speeds average diameter graph.

It can be seen from Figure 5 that as the liquid supply rate increases, the average diameter of the prepared facial mask fibers shows an increasing trend. This trend may be due to the fact that as the liquid supply rate increases, the solution flow rate also increases during the same time. However, the electric field strength is not enough to fully affect the jet flow, resulting in multi-jet instability, thereby causing an increase in fiber diameter and standard deviation. On the other hand, the liquid supply speed also affects the production efficiency of electrospinning. Therefore, taking production efficiency and fiber morphology into consideration, the liquid supply speed is preferably 130 mL/h.

## 6. Performance Analysis of Needleless Electrospinning Nano-Mask

### 6.1. Fourier Transform Infrared Spectroscopy (FTIR) Measurement

In order to analyze the chemical composition of the PEO/collagen peptide nanofiber facial mask, five groups of PEO/collagen peptide nanofiber facial masks with different ratios were tested using infrared spectroscopy using a NicoletIS50 Fourier transform infrared spectrometer. The number of scans was set to 32 and the resolution to 4. The data from OMNIC infrared spectrum software (OMNIC 9.7) were exported using Origin software (Origin 2021) to process and analyze the data.

### 6.2. Moisturizing Performance Experimental Design

In order to analyze the moisturizing and hydrating performance of the PEO/collagen peptide nanofiber facial mask, the SK-IV digital skin moisture detector was used to test the moisturizing performance of five groups of PEO/collagen peptide nanofiber facial masks with different ratios. The specific testing process is as follows:

1. Expose the target skin area to the air for 10 min at room temperature (25 °C), and then use an instrument to measure the moisture content and oil content of the skin at that time.
2. Apply five groups of PEO/collagen peptide nanofiber masks with different ratios evenly to the skin test site, and use a spray bottle containing deionized water to apply to the mask to melt and be absorbed by the skin. Then, gently massage the skin area, and after 10 min, measure the moisture content and oil content of the mask area. (Before each experiment, a certain period of time should be allowed to pass and the skin’s moisture content and oil content should be re-measured).
3. As a control experiment, while performing step 2, spray deionized water on the adjacent parts of the skin where the mask is applied, and massage gently. After 10 min, measure the skin moisture and oil content of the area at the same time.

### 6.3. Quick-Dissolving Performance Experimental Design

In order to study the quick-dissolving effect of the PEO/collagen peptide nanofiber mask, the PEO/collagen peptide nanofiber mask was cut into a rectangular shape, and then the mask sample was picked up with tweezers and placed in deionized water. In order to record the dissolution process of the PEO/collagen peptide nanofiber mask, a camera was used to film it. 

## 7. Experimental Results and Analysis

### 7.1. Infrared Spectral Analysis

**Figure 6:**
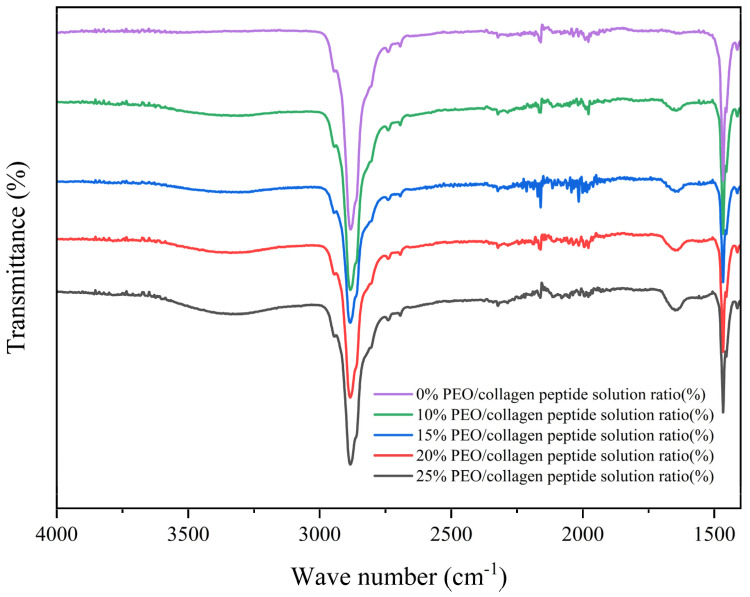
Infrared spectrum of PEO/collagen peptide nanofiber masks.

A Nicolet IS50 Fourier transform infrared spectrometer is a device that uses substances to analyze the absorption characteristics of infrared radiation of different wavelengths. This equipment can quickly analyze the chemical structure and composition of the PEO/collagen peptide nanofiber mask without damaging it. In this experiment, five groups of PEO/collagen peptide nanofiber facial masks prepared with different ratios were subjected to infrared spectrum detection. The results treated with Origin are shown in Figure 6.

As can be seen from Figure 6, after adding collagen peptide, compared with the PEO nanofiber mask, the infrared spectrum has obvious changes at 1650 cm^−1^ and 3400 cm^−1^, which correspond to the characteristic peaks of collagen peptide. Among them, the characteristic peak of C=O of (−CO−NH−) in the collagen peptide molecule was observed at 1650 cm^−1^, which is the amide I region. A characteristic peak appears at 3400 cm^−1^, corresponding to the stretching vibration of hydroxyl groups and N-H in collagen peptides. The analysis results in Figure 6 show that collagen peptides are successfully loaded into the PEO/collagen peptide nanofiber mask.

### 7.2. Moisturizing Performance Analysis

**Figure 7:**
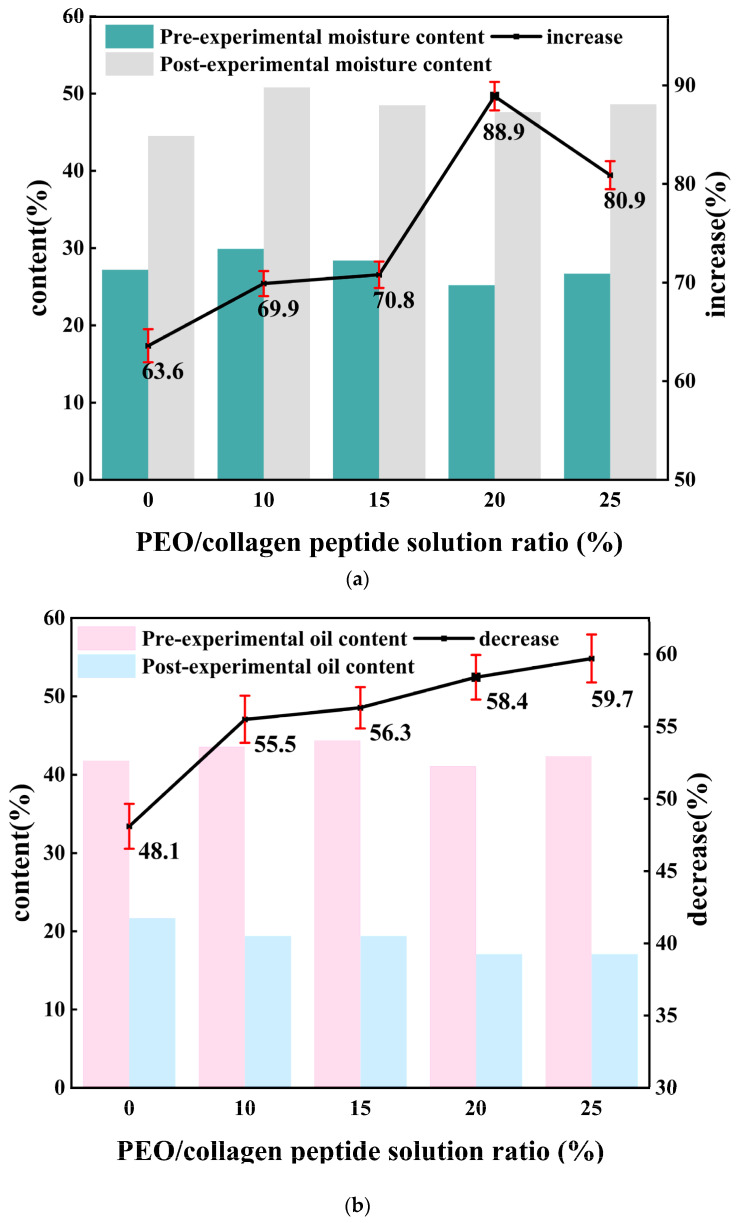
(**a**) Effects of different proportions of collagen peptide content on skin moisture; (**b**) effects of different proportions of collagen peptide content on skin oil content.

Figure 7 shows the results of testing the moisturizing performance of PEO/collagen peptide masks with different ratios. As can be seen from the table, compared with spraying ionized water directly onto the skin, applying the PEO/collagen peptide mask to the skin can significantly increase the moisture content of the skin and reduce the oil content of the skin, with better results.

As the content of the functional molecule collagen peptide loaded in the nanofiber mask increases, the skin moisture content increases by more than 60%, and the oil content decreases by more than 45%. The increase in skin moisture increased from 63.6% to 88.9%, while the decrease in oil content also increased from 48.1% to 59.7%. This shows that the moisturizing ability of the nanofiber mask is gradually enhanced, and the moisturizing effect on the skin is more obvious. Among the masks, the skin moisture of the nanofiber mask prepared with 20% collagen peptide solution increased by 88.9% and decreased by 58.4%, which was close to the 59.7% decrease of the 25% collagen peptide solution, and had better moisturizing performance. This may be because the nanofiber mask fibers prepared with 20% collagen peptide solution have the smallest average fiber diameter, are relatively uniform, and have the characteristics of high porosity and a large specific surface area. After spraying deionized water, the collagen peptides can be dissolved and absorbed faster. The skin absorbs them, resulting in enhanced moisturizing properties at this time. In addition, in this experiment, the nanofiber mask underwent rigorous skin testing and proved to be non-irritating and non-inflammatory to the skin.

### 7.3. Quick-Dissolving Performance Analysis

**Figure 8:**
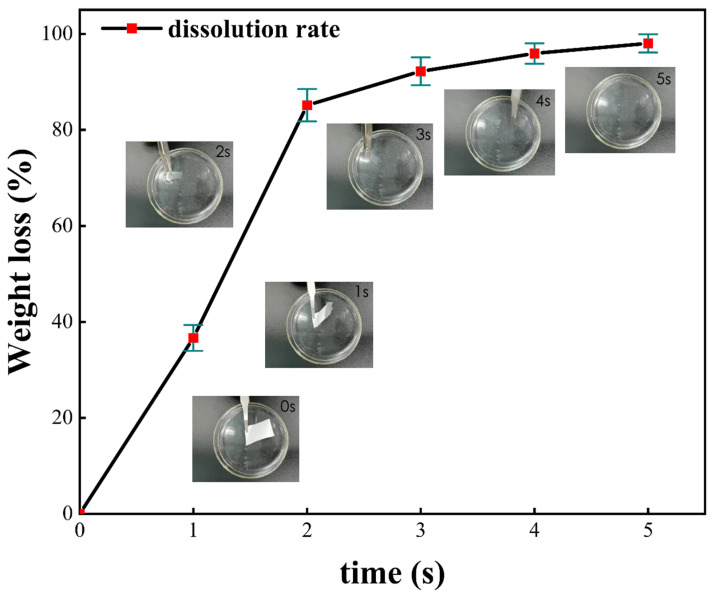
Quick-dissolving effect of PEO/collagen peptide nanofiber mask.

The quick-dissolving dissolution experimental process of the PEO/collagen peptide nanofiber mask is shown in Figure 8. The time in the upper right corner indicates the dissolution of the nanofiber mask at this time. It can be clearly seen that the PEO/collagen peptide nanofiber mask sample begins to dissolve the moment it is put into the ionized water, and quickly spreads around. Within 3 s, most of the main body of the mask disappeared, visible to the naked eye, leaving some tiny particles floating in the water. At this time, the dissolution rate gradually slowed down. At 5 s, the fiber particles had completely dissolved. During the whole process, it can be clearly seen that the PEO/collagen peptide nanofiber mask dissolves very quickly, and as time goes by, the dissolution rate gradually decreases.

The reason why the PEO/collagen peptide nanofiber mask can dissolve quickly is related to the following factors: First of all, the carrier component PEO of the PEO/collagen peptide nanofiber mask and the functional component collagen peptide are both hydrophilic, making the PEO/collagen peptide nanofiber mask easily soluble in water. Secondly, the electrospun fiber has the advantages of a large specific surface area and high porosity, which makes the contact area between the fiber and water larger and makes it easier to dissolve in water.

Therefore, the PEO/collagen peptide nanofiber mask is a quick-dissolving nanofiber mask. A unique advantage of this solid-state nanofiber mask is that after using a spray bottle, spraying water or essence, the water-soluble polymer can be quick-dissolving, allowing the small molecules to be quickly and efficiently absorbed. It is easy to use and can save a lot of time; the quick-dissolving process is visualized, and has obviously visual effects.

## 8. Discussion

In this article, a new type of PEO/collagen peptide nanofiber, quick-dissolving facial mask was prepared based on needleless electrospinning technology. During the production process, multiple Taylor cones are used to form dense multi-jet streams, which effectively avoids the defects of traditional electrospinning. The jets formed spontaneously on the surface of the silk electrode greatly increase the output of the silk electrode and realize wthe industrialization of nanofiber facial masks. The influence of different process parameters on the micromorphology of nanofiber facial masks was also explored, including the influence of different mixing ratios of PEO/collagen peptide solutions, spinning voltage, collection distance, and liquid supply speed on the mask morphology. Through research, the optimal process parameters for preparing nanofiber masks were obtained, five groups of PEO/collagen peptide nanofiber masks with different ratios were prepared, and the composition, moisturizing performance and quick-dissolving effect of the PEO/collagen nanofiber masks were comprehensively analyzed. The result is a high-performance nanofiber mask that is preservative-free, has effective molecules that are quickly absorbed by the skin, and is easy to use.

According to the single-factor experiment, it is concluded that: When the spinning liquid is 20% collagen peptide solution, the spinning voltage is 30 kV, the collection distance is 19 cm, and the liquid supply speed is 130 mL/h, the mask morphology is optimal. Five groups of PEO/collagen peptide nanofiber masks with different ratios were tested for the infrared spectrum. It was found that the infrared spectrum had characteristic peaks corresponding to collagen peptides, proving that collagen peptides exist in the nanofiber masks. Then, the moisturizing performance of the five groups of PEO/collagen peptide nanofiber facial masks with different ratios was tested. The results showed that as the amount of collagen peptide added increased, the moisturizing performance became stronger. Finally, the quick-dissolving effect of the solid-state nanofiber mask was explored. The mask was completely dissolved after being soaked for 5 s, and the dissolution speed was extremely fast. Compared with traditional facial masks, nanofiber facial masks have more advantages in preservative addition, essence adsorption capacity, compatibility, breathability, and production cost, and are more cost-effective.

## Data Availability

The original contributions presented in the study are included in the article, further inquiries can be directed to the corresponding authors.
